# The PadR-like transcriptional regulator LftR ensures efficient invasion of *Listeria monocytogenes* into human host cells

**DOI:** 10.3389/fmicb.2015.00772

**Published:** 2015-07-28

**Authors:** Karan G. Kaval, Birgitt Hahn, Nayana Tusamda, Dirk Albrecht, Sven Halbedel

**Affiliations:** ^1^FG11 Division of Enteropathogenic Bacteria and Legionella, Robert Koch InstituteWernigerode, Germany; ^2^Institute of Microbiology, University of GreifswaldGreifswald, Germany

**Keywords:** multi drug resistance, circadian rhythm, swarming, ethidium bromide uptake, repressor proteins

## Abstract

Invasion of the bacterial pathogen *Listeria monocytogenes* into human host cells requires specialized surface molecules for attachment and induction of phagocytosis. However, efficient invasion is also dependent on factors with house-keeping functions, such as SecA2-dependent secretion of autolysins for post-divisional segregation of daughter cells. Mutations in this pathway prevent degradation of peptidoglycan cross-walls, so that long cell chains are formed that cannot be phagocytosed. The extreme chaining of such mutants manifests as rough colony phenotype. One rough clone was isolated from a transposon library with a transposon insertion in the uncharacterized *lmo0720* gene (*lftS*) together with a spontaneous point mutation in the *secA2* gene. We separated both mutations and demonstrated that this point mutation in the intramolecular regulator 2 domain of SecA2 was sufficient to inactivate the protein. In contrast, *lftS* deletion did not cause a Δ*secA2*-like phenotype. *lftS* is located in an operon with *lftR* (*lmo0719*), encoding a PadR-like transcriptional regulator, and *lftR* deletion affected growth, invasion and day-light dependent coordination of swarming. Inactivation of *lftS* partially suppressed these phenotypes, suggesting a functional relationship between LftR and LftS. However, the invasion defect of the Δ*lftR* mutant was only marginally suppressed by *lftS* removal. LftR regulates expression of the *lmo0979–0980* (*lieAB*) operon, encoding a putative multidrug resistance transporter and *lieAB* transcription was strongly upregulated in the absence of LftR. Deletion of *lieAB* in the Δ*lftR* background restores wild type-like invasion levels. Hence, we conclude that tight transcriptional repression of the *lieAB* operon is essential for efficient listerial host cell invasion.

## Introduction

*Listeria monocytogenes* is an opportunistic pathogen, which can cause life-threatening gastrointestinal infections in humans upon ingestion of contaminated food. Cells of *L. monocytogenes* can actively invade non-phagocytic host cells and persist and multiply inside their cytoplasm (Freitag et al., 2009). Motility inside the host cell is facilitated by comet tail-like polymerization of host actin at one bacterial cell pole that generates the driving force to push the bacteria through the viscous cytoplasm (Tilney and Portnoy, 1989; Dabiri et al., 1990; Lambrechts et al., 2008). The same mechanism allows generation of membranous protrusions at the surface of the infected host cell, which are internalized by neighboring cells and finally mediates listerial spread from cell to cell (Tilney and Portnoy, 1989; Ireton et al., 2014). Using this strategy, *L. monocytogenes* manages to spread within host tissues and breaches all barriers of the human body, i.e., the gastrointestinal, the placental and the blood brain barrier, thereby causing gastrointestinal symptoms and infections of the brain or the fetus (Cossart and Toledo-Arana, 2008). Thus, invasion is the very first step in a sequence of events that eventually lead to manifestation of listeriosis, which, remarkably, can cause case-fatality rates of up to 30% (Swaminathan and Gerner-Smidt, 2007; Hsieh et al., 2009).

Listerial invasion depends on internalins, which are specialized bacterial surface proteins that contact receptors at the host cell surface. *L. monocytogenes* encodes 25 internalins (Gaillard et al., 1991; Dramsi et al., 1995; Bierne et al., 2007). However, the most prominent members of this protein class are InlA and InlB, which bind to E-cadherin and Met (Mengaud et al., 1996; Shen et al., 2000), respectively, at the host cell surface and these protein–protein interactions induce cytoskeletal re-arrangements in the host cells that lead to uptake of the bacterium in a phagocytosis-like process (Cossart and Toledo-Arana, 2008). Apart from these invasion-specific molecules, mutations in other genes with more general house-keeping functions have been shown to severely reduce the invasive potential of *L. monocytogenes*. Among these, factors contributing to synthesis, modification and degradation of the bacterial envelope are predominant (Seveau et al., 2007; Camejo et al., 2011; Pizarro-Cerda et al., 2012).

Here we characterize the functions of four so far unknown genes of this latter type that encode for obvious house-keeping functions but affect invasion. This includes the *lmo0719–0720* genes, which are organized in a bi-cistronic operon. While *lmo0720* codes for a gene of unknown function, *lmo0719* shares homology with PadR-type transcriptional regulators. PadR-like transcriptional regulators can be found in many bacteria and are often associated with control of detoxification genes (Barthelmebs et al., 2000; Gury et al., 2004; Agustiandari et al., 2008). In contrast, genes homologous to *lmo0720* are less wide-spread and are specific to the genus *Listeria* and a few other Gram-positive bacteria. We demonstrate that Lmo0719 controls transcription of an uncharacterized putative ABC multidrug resistance transporter, encoded by the *lmo0979–0980* operon, which shares similarity with the LmrCD transporter of *Lactococcus lactis* (Lubelski et al., 2006). Strikingly, invasion of Δ*lmo0719* mutants into HeLa cells was strongly reduced and hence, *lmo0719* was renamed *lftR* (listerial protein facilitating invasion/transcriptional regulator, and accordingly, *lmo0720* was renamed *lftS*). This defect could be restored by deletion of the *lmo0979–0980* operon, suggesting that tight repression of *lmo0979–0980* transcription through LftR is necessary to ensure efficient invasion of eukaryotic host cells. Further experiments identified ethidium bromide as one artificial substrate, which is taken up by this transporter, and thus the *lmo0979–0980* genes were renamed *lieAB* (listerial importer of ethidium bromide as artificial substrate).

## Materials and Methods

### Bacterial Strains and Growth Conditions

All strains used in this study are listed in **Table 1**. Cells of *L. monocytogenes* were generally cultivated in BHI broth or on BHI agar plates at 37°C and 200 rpm if not stated otherwise. Where required, antibiotics and supplements were added at the following concentrations: erythromycin (5 μg/ml), kanamycin (50 μg/ml) and X-Gal (100 μg/ml). *Escherichia coli* TOP10 was used as standard cloning host (Sambrook et al., 1989).

**Table 1:** Strains and plasmids used in this study.

Name	Relevant characteristics	Reference^∗^
**Plasmids**		
pET11a	*bla* P*_*T7*_ lacI*	Novagen
pIMK2	P*_*help*_ neo*	Monk et al. (2008)
pIMK3	P*_*help*_-lacO lacI neo*	Monk et al. (2008)
pMAD	*bla erm bgaB*	Arnaud et al. (2004)
pMC38	mini transposon delivery vector	Cao et al. (2007)
pUC19	*bla lacZα*	Invitrogen
pSH314	*bla erm bgaB* Δ*secA2*	Halbedel et al. (2012)
pKK36	P*_*help*_-secA2 neo*	This work
pKK37	*bla*Δ*lftS*	This work
pKK38	P*_*help*_-secA2G484E neo*	This work
pKK39	*bla erm bgaB* Δ*lftS*	This work
pKK40	*bla erm bgaB* Δ*lftRS*	This work
pKK43	P*_*help*_-lacO-lftR lacI neo*	This work
pKK53	*bla lftR genomic region*	This work
pKK54	*bla*Δ*lftR*	This work
pKK56	*bla erm bgaB* Δ*lftR*	This work
pKK64	*bla erm bgaB lftS::Tn*	This work
pNT1	P*_*help*_-lieAB neo*	This work
pNT3	P*_*help*_-lieA(K44E)lieB neo*	This work
pSH346	*bla* P*_*T7*_-secA2-his_6_ lacI*	This work
pSH348	*bla* P*_*T7*_-secA2-strep lacI*	This work
pSH399	*bla erm bgaB* Δ*lieAB*	This work
***Listeria monocytogenes* strains**		
EGD-e	wild type, serovar 1/2a strain	Glaser et al. (2001)
LMS81	Δ*secA2*	Halbedel et al. (2012)
LMKK18	*lmo0720::Tn secA2G484E*	This work
LMKK24	*attB::*P*_*help*_-secA2 neo*	pKK36 → EGDe
LMKK25	*attB::*P*_*help*_-secA2G484E neo*	pKK38 → EGDe
LMKK26	*ΔlftS* (*lmo0720*)	pKK39 ↔ EGDe
LMKK27	Δ*secA2 attB::*P*_*help*_-secA2 neo*	pSH314 ↔ LMKK24
LMKK28	Δ*secA2 attB::*P*_*help*_-secA2G484E neo*	pSH314 ↔ LMKK25
LMKK31	*ΔlftRS* (*lmo0719-0720*)	pKK40 ↔ EGDe
LMKK42	*ΔlftR* (*lmo0719*)	pKK56 ↔ EGDe
LMKK62	Δ*lftR attB::*P*_*help*_-lacO-lftR lacI neo*	pKK43 → LMKK42
LMKK64	*lftS::Tn*	pKK64 ↔ EGDe
LMNT1	*attB::*P*_*help*_-lieAB neo*	pNT1 → EGDe
LMNT2	*attB::*P*_*help*_-lieA(K44E)lieB neo*	pNT3 → EGDe
LMS160	Δ*lieAB* (*lmo0979-lmo0980*)	pSH399 ↔ EGD-e
LMS168	Δ*lftRS* Δ*lieAB*	pSH399 ↔ LMKK31
LMS169	Δ*lftR* Δ*lieAB*	pSH399 ↔ LMKK42

*^∗^The arrow (→) stands for a transformation event and the double arrow (↔) indicates gene deletions obtained by chromosomal insertion and subsequent excision of pMAD plasmid derivatives (see experimental procedures for details).*

### General Methods, Oligonucleotides, and Manipulation of DNA

Transformation of *E. coli*, isolation of plasmid and chromosomal DNA was performed using standard methods (Sambrook et al., 1989). Preparation of electro-competent *L. monocytogenes* cells and their transformation were carried out as described elsewhere (Monk et al., 2008). Restriction and ligation of DNA was done as per the manufacturer’s instructions. For restriction free modification of plasmids an altered version of the original Quickchange mutagenesis protocol was employed (Zheng et al., 2004). All primer sequences are listed in **Table 2**.

**Table 2:** Oligonucleotides used in this study.

Name	Sequence (5′→3′)
SHW40	GCATGCCATGGAGACTTTGATTTGCTCTGCTTC
SHW134	GCATGCCATGGAAGCTAGTAACTATGGTAGAATG
SHW241	CGCGGATCCTTATGTTGGTGCAACTGGAGTGC
SHW334	TAATAATCGCTGGTGTAATCGC
SHW311	AAAACTGCAGAGACAGAATTATGATGATCG
SHW399	GCGCACTAGTAGACAGAATTATGATGATCGAAA
SHW400	CGCGCTCGAGTTAGTGATGGTGATGGTGATGGCCTTGGATTAAGCCGTCTGG
SHW401	GCGCACTAGTCATATGTATATCTCCTTCTTAAAG
SHW402	CGCGCTCGAGCAAAGCCCGAAAGGAAGCTG
SHW421	TTTGCTCGAGTTATTTTTCGAACTGCGGGTGGCTCCAGCCTTGGATTAAGCCGTCTGG
SHW422	TAACTCGAGCAAAGCCCGAAAGGAAGCTG
SHW427	GTGAAATACCGCACAGATGC
SHW428	GGCATCCGCTTACAGACAAG
SHW437	CTGGTCGGGAAACGGATATCAAACTGG
SHW438	GATATCCGTTTCCCGACCAGCCATGTTCG
SHW520	GCAGCAAGATCTTTTTCTGTTCACCAGTTGGTCC
SHW521	ATATGTCGACCGAAAAACGTGCAAAAGATCCG
SHW526	AATGGGATCCTAAAATAAAAAAGGTTGGCTCCGC
SHW527	TTTAGGATCCCATTTGAATACAACCTTCTTTCC
SHW626	CGCGCGGGTACCTTATACCATTTTTTTATAAATAGTTACTGC
SHW627	CGCGCGCCATGGAAGAAGTGATGATTAAGGCC
SHW628	GTGCGGGCGAAACGACAACCATCCAAATTTTAG
SHW629	GTTGTCGTTTCGCCCGCACCGTTTGAGCC
SHW630	CTAATACGACTCACTATAGGGAGAATTCAACTTCAGCAGGTGGG
KK42	ATATGTCGACGCAAGCCAACTTCAAAACATAG
KK43	GCATGCCATGGAAGGCAAGGTGGTGATCAAAG
KK44	GTAATGGGATCCTAAAAAAACAGAAAGCCTCTCAAAA
KK45	TTTAGGATCCCATTACGCTTGCCCTCCTTTAAC
KK53	ACGCGTCGACTTAGCCTTGGATTAAGCCGTCTGG
KK56	TTTTAGGATCCTTAGTAGCCGTATGTTTCTCCTC
KK57	TACTAAGGATCCTAAAAAAACAGAAAGCC
KK66	CATGCCATGGATGAAAGGACTTACCGAGTTACTC
KK67	ATATGTCGACTTACGCTTGCCCTCCTTTAACTTG
KK75	CCTCCGCTAGCCATTTGAATTCACTCCTCTACTAG
KK76	AAATGGCTAGCGGAGGGCAAGCGTAATGTTTAATTG

### Transposon Mutagenesis and Inverse PCR

The *HimarI* transposon delivery vector pMC38 (Cao et al., 2007) was introduced into *L. monocytogenes* by electroporation and erythromycin-resistant clones were selected at 30°C. Five colonies were randomly chosen and grown in BHI medium supplemented with kanamycin and erythromycin at 30°C overnight. These cultures were diluted 1:200 in fresh BHI broth containing erythromycin and incubated at 30°C for 1 h. Afterward, the temperature was increased to 42°C for 6 h until an OD_600_ of 0.5 was reached. These cultures were serially diluted and plated on both BHI erythromycin as well as BHI kanamycin plates to determine CFU/ml and rate of plasmid retention. Aliquots were mixed with 50% glycerol and frozen at -80°C.

Rough colonies were isolated and plated on BHI agar containing erythromycin and BHI agar plates containing kanamycin. Chromosomal DNA was isolated from *erm^R^ kan^S^* clones, which were then screened for transposon insertions in *secA2* or *divIVA* as determined by PCR using the primer pairs SHW241/SHW334 and SHW134/SHW40, respectively. The site of transposon insertion was determined by inverse PCR. For this purpose, chromosomal DNA of relevant clones was subjected to TaqI digestion for 1 h, followed by a 20 min heat inactivation step. Ligation of the resultant digested DNA was carried out for 1 h at room temperature using T4 DNA ligase. A PCR was set up using the primer pair SHW427/SHW428 and the ligation mixture as the template. This PCR product was purified and the transposon insertion site was determined by DNA sequencing.

### Construction of Plasmids and Strains

In order to facilitate overexpression of SecA2-Strep, plasmid pSH348 was constructed. It was obtained in two steps. In the first step, the *secA2* gene was amplified using SHW399/SHW400 (SHW400 introduced a C-terminal His_6_-tag) and the resulting fragment was digested with SpeI/XhoI and cloned into pET11a, which had been linearized using the primer pair SHW401/SHW402. The His_6_-tag present at the C-terminus in the resulting plasmid (pSH346) was then replaced by a C-terminal Strep-tag in a PCR using SHW421/SHW422 as the primers.

Plasmid pKK36 was constructed to allow for constitutive expression of the *secA2* gene in *L. monocytogenes*. It was obtained by amplification of the *secA2* gene using the primer pair SHW311/KK53. The resulting fragment was PstI/SalI cut and ligated with the similarly cut backbone of pIMK2. The G484E mutation was brought into this vector by quickchange mutagenesis using the oligonucleotides SHW437/SHW438, yielding plasmid pKK38.

Plasmid pKK39, allowing marker-less removal of the *lftS* gene was obtained in two steps: first, up- and downstream regions of the *lftS* gene were amplified in PCRs with the primer pairs KK42/KK45 and KK44/KK43, respectively. Both fragments were joined together by ligation after their ends had been made compatible by BamHI digestion. The desired Δ*lftS* fragment was then amplified from the ligation mixture in a second PCR using the primer pair KK42/KK43 and blunt end cloned into SmaI cut pUC19. The Δ*lftS* fragment of the resulting plasmid (pKK37) was finally sub-cloned into pMAD using NcoI/SalI restriction digestion. Plasmid pKK40 for deletion of the entire *lftRS* operon was obtained by deletion of the *lftR* gene from plasmid pKK39 using the primer pair KK56/KK57.

For deletion of the *lftR* gene, plasmid pKK56 was constructed in three steps. First, the chromosomal region encompassing the *lftR* gene was amplified with the oligonucleotides KK42/KK43 and blunt end cloned into SmaI cut pUC19. The *lftR* gene was then removed from the resulting plasmid (pKK53) by PCR using the primer pair KK75/KK76, which yielded plasmid pKK54. Finally, the NcoI/SalI digested Δ*lftR* fragment of this plasmid was sub-cloned into similarly cut pMAD.

Plasmid pKK64 was generated to replace the *lftS* gene of strain EGD-e by the *lftS*::*Tn* allele of the rough transposon insertion mutant LMKK18. For this purpose, the *lftS::Tn* fragment was amplified by PCR from LMKK18 chromosomal DNA using the primer pair KK42/KK43, NcoI/SalI cut and ligated with the backbone of pMAD digested with the same enzymes.

In order to remove the *lieAB* operon from the listerial chromosome, plasmid pSH399 was designed. Up- and downstream regions of the *lieAB* operon were amplified with the primer pair SHW520/SHW527 and SHW526/SHW521, respectively, BamHI cut and fused together by ligation. The desired Δ*lieAB* fragment was amplified from the ligation mixture in a PCR using SHW520/SHW521 as the primers and cloned into pMAD using BglII/SalI.

For IPTG-inducible expression of *lftR*, plasmid pKK43 was constructed. The *lftR* gene was amplified in a PCR using KK66/KK67 as the primers and the resulting fragment was cloned into pIMK3 via NcoI/SalI.

Plasmid pNT1 was constructed for overexpression of *lieAB*. It was obtained by amplification of the *lieAB* operon using the primer pair SHW627/SHW626 and cloned with NcoI/KpnI into pIMK2. The K44E mutation was brought into the Walker motif of the *lieA* gene on plasmid pNT1 in a quickchange reaction with the primer pair SHW628/SHW629. The resulting plasmid was named pNT3.

pIMK2 and pIMK3 plasmids were introduced into *L. monocytogenes* strains by electroporation and kanamycin resistant clones were selected. Plasmid insertion at the *attB* site of the tRNA^Arg^ locus was verified by PCR. For the marker-less removal of genes, pMAD derivatives were transformed into the respective *L. monocytogenes* recipient strains and the genes were removed according to a previously described protocol (Arnaud et al., 2004). All gene deletions were verified by PCR.

### Purification of SecA2 and Generation of an Anti-SecA2 Antiserum

SecA2-Strep was overexpressed in *E. coli* BL21. The bacteria were cultivated in LB broth containing ampicillin (100 μg/ml) at 37°C. Expression of the SecA2 protein was induced at an optical density of OD_600_ = 0.5 by addition of 1 mM IPTG (final concentration) after the culture had been cooled down to 18°C. Cultivation was continued over night at 18°C and cells were harvested, washed once with ZAP buffer (10 mM Tris/HCl pH 7.5 and 200 mM NaCl), and finally disrupted in the same buffer using the emulsiflex homogenisator system (Avestin, Germany). Cell debris were removed from the lysate by centrifugation (6000 × *g*, 5 min, 4°C) and the supernatant was ultracentrifuged (100.000 × *g*, 30 min 4°C). Strep-tagged proteins were then purified from the cleared lysates using affinity chromatography and Strep-Tactin^®^ sepharose (IBA Lifesciences, Germany). Elution fractions containing SecA2-Strep were stored at -20°C. Purified SecA2-Strep was used for immunization of one rabbit to generate a polyclonal antiserum recognizing SecA2 (Seqlab, Germany).

### Isolation of Cellular Proteins and Protein Detection Techniques

Cells were harvested by centrifugation (6000 × *g*, 5 min, 4°C) and washed once with ZAP buffer. The cell pellet was resuspended in 1 ml ZAP buffer also containing 1 mM PMSF and disrupted by sonication. Cell debris were removed by centrifugation and the resulting supernatant was considered as total cellular protein extract. Aliquots of these samples were separated by standard sodium dodecyl sulfate polyacrylamide gel electrophoresis (SDS PAGE). Gels were either stained using the colloidal coomassie agent Roti^®^-Blue (Roth, Germany) or transferred onto positively charged polyvinylidene fluoride (PVDF) membranes using a semi-dry transfer unit. Proteins of interest were immune-stained using polyclonal rabbit antisera recognizing DivIVA (Marston et al., 1998) or SecA2 (this work) as the primary antibody and anti-rabbit immunoglobuline G conjugated to horseradish peroxidase as the secondary one. The ECL chemiluminescence detection system (Thermo Scientific) was used for detection of the peroxidase conjugates on the PVDF membrane in a chemiluminescence imager (Vilber Lourmat). Protein identification by mass spectroscopy was performed as described recently (Halbedel et al., 2014).

### RNA Isolation and Northern Blotting

Bacteria were cultivated in BHI broth at 37°C and total RNA was extracted from cells obtained from 5 ml cultures grown to an optical density of 0.8 (λ = 600 nm) using the RNeasy Mini Kit (Qiagen). Northern blot analysis was performed as described by Wetzstein et al. (1992). The *lieA*-specific digoxigenin-labeled RNA probe was generated by *in vitro* transcription with T7 RNA polymerase (Roche Diagnostics) and a *lieA*-internal PCR fragment generated with the primer pair SHW627/SHW630 (the reverse primer introduced the T7 RNA polymerase recognition site). *In vitro* transcription was carried out using the DIG RNA labeling Kit (Roche). Hybridisation and signal detection were performed using the DIG wash and block buffer set, an anti-digoxigenin antibody conjugated to alkaline phosphatase and the CDP-Star reagent (all chemicals obtained from Roche) according to the manufacturer’s instructions.

### Swarming Assays

LB soft agar plates containing 0.3% agar were stab inoculated with the respective strains of *L. monocytogenes*. Plates were incubated at 30°C for 24 h to observe swarming halos. In order to observe concentric ring formation in swarming halos associated with the circadian swarming rhythm, the plates were kept for an additional 6 days at room temperature and thus exposed to a natural course of daylight.

### Drug Susceptibility Assays

Determination of minimal inhibitory concentrations was performed as described earlier (Rismondo et al., 2015). MIC test strips with the following concentration gradients were purchased from Bestbiondx (Germany): Tetracycline (0.016–256 μg/ml), gentamicin (0.016–256 μg/ml), chloramphenicol (0.016–256 μg/ml) and vancomycin (0.016–256 μg/ml). Filter disk assays were used for susceptibility tests against ethidium bromide and Hoechst 33342. Sterile whatman paper disks (Ø 6 mm) were soaked with 27 μl of a 1 mg/ml ethidium bromide solution or a solution containing 10 mg/ml Hoechst 33342 and laid on top of BHI agar plates, which had been swab-inoculated with a re-suspension of the *L. monocytogenes* strains to be tested. Zones of growth inhibition became visible after overnight incubation at 37°C.

### Ethidium Bromide Uptake Assay

Ethidium bromide was used as model substrate for the LieAB transporter. Measurement of ethidium bromide uptake was performed as described elsewhere with minor modifications (Neyfakh et al., 1991). Briefly, *L. monocytogenes* strains were grown in BHI broth at 37°C until an OD_600_ of about 0.5 was reached. Cell were washed once in phosphate buffered saline (PBS), resuspendend in PBS to a final OD_600_ of 0.5 and 100 μl aliquots were pipetted into the wells of a black 96-well plate. Ethidium bromide was added to a final concentration of 5 μg/ml and fluorescence was measured over time in a Tecan infinite M1000 microplate reader (λ_excitation_ = 500 nm, λ_emission_ = 580 nm).

### Microscopy

Samples (0.4 μl) from exponentially growing cultures were spotted onto microscope slides, covered with a thin agarose film (1.5% in distilled water). Samples were air-dried, covered with a cover lid and subjected to phase contrast or fluorescence microscopy. Staining of membranes was performed by addition of nile red (1 μg/ml final concentration) to 100 μl of culture and shaking for 20 min at 37°C. Images were taken with a Nikon Eclipse Ti microscope coupled to a Nikon DS-MBWc CCD camera. Bacterial colonies were imaged with an inverse Nikon Diaphot 300 microscope connected to a Digital Sight DS-Fi1 camera. Images were processed using the NIS elements AR software package (Nikon).

### Cell Culture Techniques

Experimental infections of HeLa cells and J774 mouse macrophages were performed as described earlier (Halbedel et al., 2012, 2014).

## Results

### Identification of the *lftRS* Genes

**FIGURE 1:**
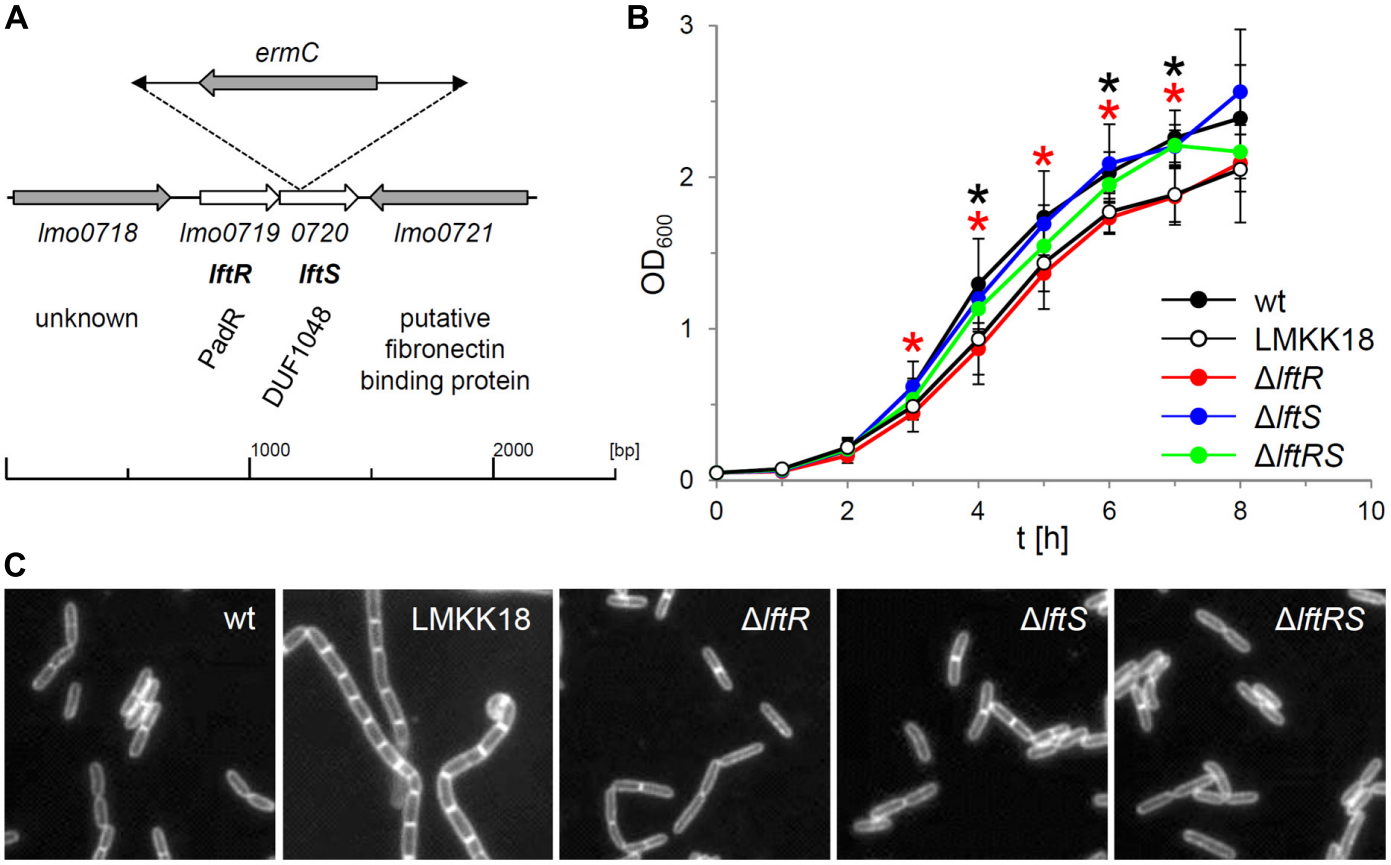
**Growth phenotypes of *lftRS* mutant strains. (A)** Schematic representation of the site of transposon insertion in the genome of strain LMKK18. Please note that the *ermC* gene located in the mini-transposon is oriented in the direction opposite to the *lmo0719–0720* operon. **(B)** Growth of strains EGD-e (wt), LMKK18 (rough isolate, *lftS::Tn secA2G484E*), LMKK42 (Δ*lftR*), LMKK26 (Δ*lftS*) and LMKK31 (Δ*lftRS*) in BHI broth at 37°C. Values are averages from 5 repetitions. SD are indicated. Significance levels (*P* < 0.05) are indicated for strains LMKK18 (*lftS::Tn secA2G484E*, black asterisks) and LMKK42 (Δ*lftR*, red asterisks). **(C)** Micrographs showing nile red stained cells of the same set of strains cultivated in BHI broth at 37°C up to mid-logarithmic growth phase.

From a transposon mutagenesis screen, which initially aimed at the identification of mutants with a rough colony phenotype, we identified one rough isolate without transposon insertions in either *secA2* or *divIVA*, the deletion of which typically results in the formation of rough colonies (Lenz and Portnoy, 2002; Halbedel et al., 2012). This clone was designated LMKK18 and inverse PCR revealed that the transposon had integrated into the 29th amino acid codon of the *lftS* gene (*lmo0720*) encoding a protein of unknown function with the uncharacterized DUF1048 domain (**Figure 1A**). The *lftS* ORF is part of a bicistronic operon (Toledo-Arana et al., 2009) also containing the *lftR* gene (*lmo0719*) that encodes a putative transcriptional regulator. DNA sequencing revealed that the *divIVA* gene of strain LMKK18 had the wild type sequence, whereas a point mutation in its *secA2* gene changed the glycine at position 484 into glutamate (Supplementary Figure S1A). Strain LMKK18 showed a slight but significant growth defect in BHI broth at 37°C (**Figure 1B**) and grew as long cell chains as it is characteristically observed with Δ*secA2* mutant strains (**Figure 1C**; Lenz and Portnoy, 2002; Kaval et al., 2014).

The G484 residue of *L. monocytogenes* SecA2 corresponds to G490 in *Bacillus subtilis* SecA and lies within the IRA2 domain that modulates ATP binding (Sianidis et al., 2001; Supplementary Figure S1B). This residue makes contacts with the α-phosphate of an ADP molecule (Hunt et al., 2002; Osborne et al., 2004; Supplementary Figure S1B) and should affect binding of ATP to SecA2, when mutated. In order to test this hypothesis, a complementation assay was established to analyze activity of *secA2* mutant alleles. While the Δ*secA2* mutant grew as long chains of cells and formed rough colonies on BHI agar plates, the complemented strain (LMKK27) behaved like wild type (Supplementary Figures S1C,D). In contrast, strain LMKK28, expressing the mutated SecA2G484E protein, formed cell chains and rough colonies that were indifferent from the Δ*secA2* mutant (Supplementary Figures S1C,D) although SecA2G484E was expressed (Supplementary Figure S1E). Therefore, the presence of the *secA2G484E* mutation in strain LMKK18 explains the rough phenotype of this transposant.

### Phenotype of *lftRS* Mutant Strains

We reasoned that the *secA2G484E* mutation might have arisen as a suppressor mutation in response to the transposon insertion in the *lftS* gene itself. In order to address this question, mutant strains lacking the *lftR* (LMKK42), the *lftS* (LMKK26) or the whole *lftRS* operon (LMKK31) were generated. These mutants were grown in BHI broth and analyzed by microscopy. This showed that all three mutant strains grew as wild type-like rods in contrast to strain LMKK18, which formed a chain of cells (**Figure 1C**). Cell length measurements of roughly 300 cells per strain furthermore demonstrated that neither LMKK18 nor any of the *lftRS* deletion mutants suffered from defective cell division (data not shown). During these experiments we repeatedly observed that strain LMKK42 (Δ*lftR*) showed the same slight but significant growth defect as the rough transposon mutant LMKK18 (**Figure 1B**), while the Δ*lftS* mutant (LMKK26) grew as fast as the wild type strain EGD-e and strain LMKK31, lacking the entire *lftRS* operon, grew with an intermediate kinetic (**Figure 1B**). Thus, growth of the rough transposon isolate LMKK18 is apparently identical to that of the Δ*lftR* deletion mutant. This indicates that the Tn insertion in *lftS* might exert a deleterious polar effect on expression of the *lftR* gene. In line with the absence of a rough phenotype in strains lacking *lftR, lftS* or both genes, no effects on expression of SecA2 or DivIVA were observed in these mutants (data not shown). In order to test the possibility whether the transposon insertion in the *lftS* gene of strain LMKK18 would generate a phenotype different from those observed with the clean deletion mutants, we replaced the *lftS* gene of strain EGD-e by the *lftS::Tn* allele of strain LMKK18. The resulting strain (LMKK64) formed smooth colonies (Supplementary Figure S2A). This shows that the *lftRS* genes are not involved in expression of the rough phenotype. Moreover, none of the constructed *lftRS* mutants was prone to spontaneous transition to the rough phenotype, also not during prolonged cultivation on BHI agar plates. Thus, we assumed that the *secA2G484E* mutation in LMKK18 occurred just by chance.

### Daylight-Dependent Control of Swarming Motility Requires LftR

**FIGURE 2:**
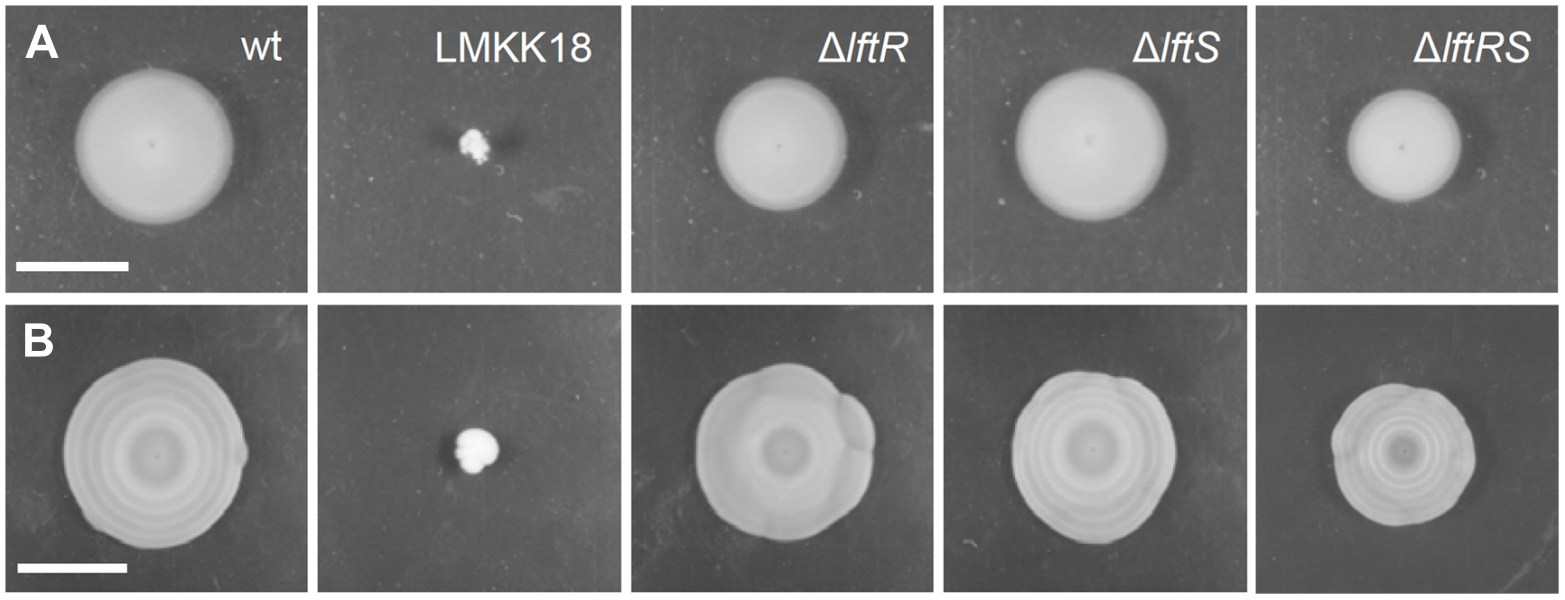
**Swarming motility of mutants lacking the *lftRS* genes. (A,B)** Swarming motility assay for EGD-e (wt), LMKK18 (*lftS::Tn secA2G484E*), LMKK42 (Δ*lftR*), LMKK26 (Δ*lftS*) and LMKK31 (Δ*lftRS*). **(A)** Soft LB agar plates were stab inoculated with the above strains from glycerol stocks and incubated at 30°C for 24 h and then documented. **(B)** The same plates photographed after 6 days of growth at room temperature under ambient light exposure. Scale bar in **(A)** indicates 1 cm, while that in **(B)** indicates 4 cm.

Mutants exhibiting the rough phenotype are unable to swarm on soft agar plates (Halbedel et al., 2012). Therefore, the strains lacking *lftR, lftS*, or *lftRS* were tested in a swarming assay. This revealed the absence of statistically significant differences in the formation of swarming halos obtained with these strains (**Figure 2A**). However, we observed that strain LMKK42 (Δ*lftR*), was almost unable to form the typical pattern of concentric rings, when cells were exposed to diurnal changes in daylight on swarming plates over several days at ambient room temperature (**Figure 2B**). These concentric rings correspond to alternating zones of opaque and translucent colony material, which are linked to the production of extracellular polymeric substance in opaque but not in translucent regions of the swarming halo (Tiensuu et al., 2013). This peculiar swarming phenotype was recently shown to be dependent on the coordinated action of the blue light receptor Lmo0799, on proteins controlling activation of the alternative sigma factor σ^B^, as well as on several other proteins from different functional categories (Tiensuu et al., 2013). In good agreement with a previous report (Tiensuu et al., 2013), formation of these concentric swarming halos is only established under ambient light conditions and cannot be observed when the experiment is repeated in the dark or under constant light exposure (Supplementary Figures S3A,B). Deletion of *lftS* had no effect on this phenotype (**Figure 2B**), and likewise, the reconstituted *lftS::Tn* strain LMKK64 formed concentric swarming rings similar to the wild type (Supplementary Figures S2B,C). Interestingly, deletion of the *lftS* gene in the Δ*lftR* background restored concentric ring formation in the Δ*lftRS* double mutant strain LMKK31 (**Figure 2B**). That deletion of *lftS* suppresses a phenotype associated with deletion of the *lftR* gene again indicates that both genes must be functionally linked.

### LftR Inhibits Expression of the LieAB Multidrug Resistance Transporter

**FIGURE 3:**
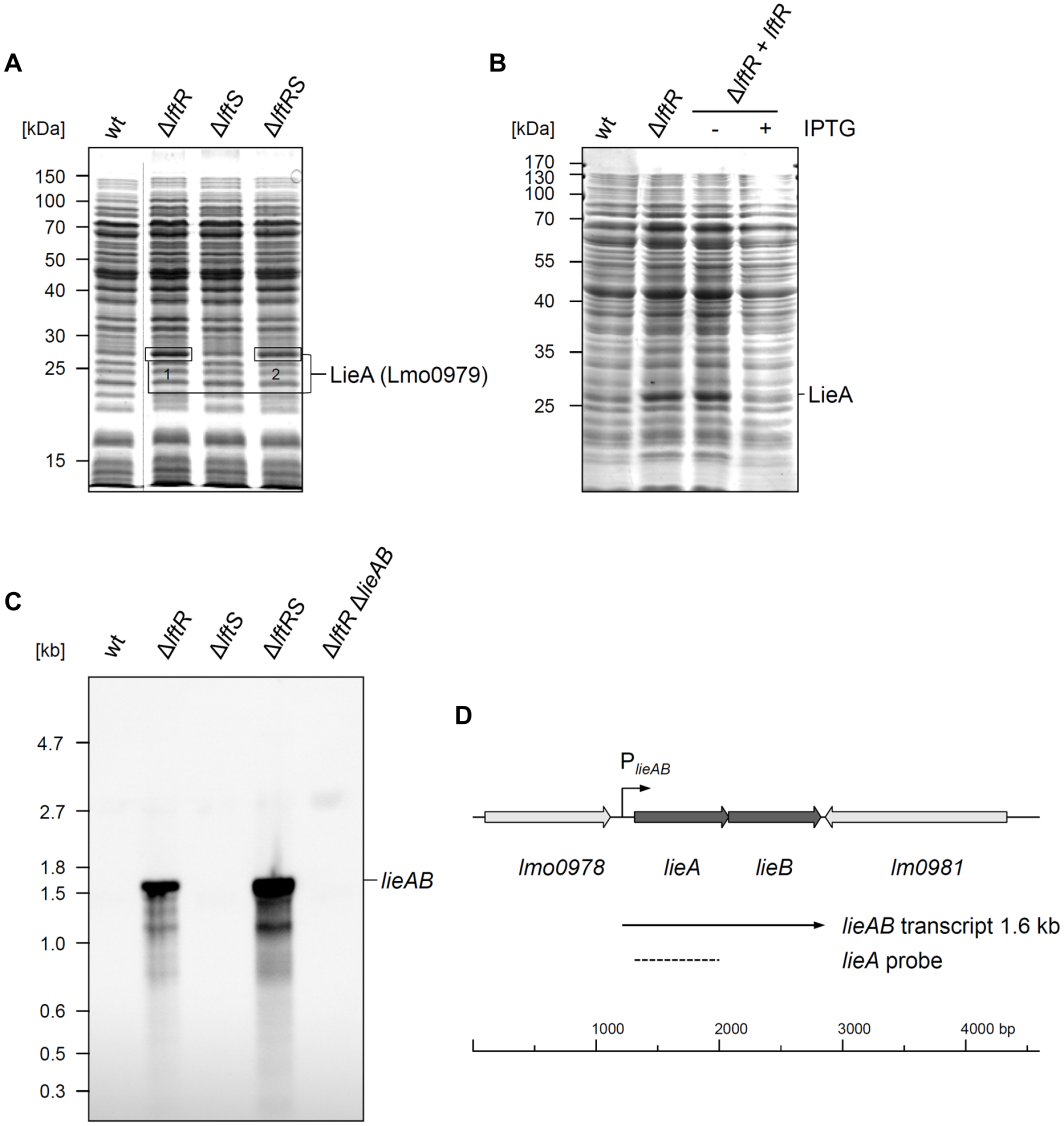
**Derepression of *lieAB* in *lftRS* mutant strains. (A)** Total cellular proteins were isolated from strains EGD-e (wt), LMKK42 (Δ*lftR*), LMKK26 (Δ*lftS*) and LMKK31 (Δ*lftRS*) that had been grown to OD_600_ = 1.0 in BHI broth at 37°C. Proteins were separated by SDS-PAGE and protein bands indicated by boxes were cut from the gel and identified by mass spectrometry. MS identification results are summarized in **Table 3**. The dashed line refers to non-relevant lanes, which were removed from the image. **(B)** Complementation of the LieA overexpression phenotype of the Δ*lftR* mutant by reintroduction of LftR. Total cellular proteins of strains EGD-e (wt), LMKK42 (Δ*lftR*) and LMKK62 (I*lftR*, I – designates IPTG-dependent alleles), grown in BHI (±1 mM IPTG), were separated by SDS-PAGE and the position of LieA is indicated. **(C)** Northern blot showing *lieAB* transcript levels in the same set of strains as in **(A)**. Additionally, strain LMS169 (Δ*lftR* Δ*lieAB*) was included as control. Total RNA was isolated from logarithmically growing cells, separated in an agarose gel and probed with a DIG-labeled ribo-probe specific for *lieA* after transfer onto a nylon membrane. **(D)** Transcriptional organization of the *L. monocytogenes lieAB* locus. Positions of the P*_lieAB_* promoter, the detected *lieAB* transcript and the *lieA* probe are indicated.

The gene product of the *lftR* gene is annotated as a PadR-like transcriptional regulator and shares varying degrees of identity with recently crystallized members of this protein family, such as the Bce3449 protein of *B. cereus* (69% identity) or LmrR of *Lactococcus lactis* (28% identity) (Supplementary Figure S4A; Madoori et al., 2009; Fibriansah et al., 2012). We wondered as to whether LftR would act as a transcriptional regulator affecting gene expression and separated total cellular protein extracts of strains lacking *lftR, lftS* or both by conventional SDS PAGE, in order to identify de-repressed genes. This uncovered one clearly overexpressed band in the Δ*lftR* mutant (**Figure 3A**). Mass spectroscopy revealed that this band corresponds to the daunorubicin resistance ATP-binding protein Lmo0979 (which we have renamed here as LieA, **Table 3**). The membrane component of this putative multidrug transporter is encoded by the *lmo0980* (*lieB*) gene, which is located in an operon together with *lieA*. LieA did not accumulate in the Δ*lftS* mutant, indicating that its up-regulation was an LftR-specific effect. In good agreement with this, LieA was still overexpressed in the Δ*lftRS* strain (**Figure 3A**). This suggests that expression of LieA is solely repressed by LftR – whether LftS is present or not. To confirm overexpression of LieA upon deletion of *lftR*, the *lieAB* operon was deleted from the Δ*lftR* mutant, resulting in strain LMS169 (Δ*lftR*Δ*lieAB*). This strain, in fact, did not show overexpression of LieA anymore (Supplementary Figure S5). This demonstrated that identification of the overexpressed protein band as LieA was correct. Importantly, expression of LftR in the Δ*lftR* background (strain LMKK62, I*lftR*, I – designates inducible alleles) from an IPTG-dependent promoter cured the LieA overexpression effect in an inducer-dependent manner (**Figure 3B**).

**Table 3:** Protein identification results.

No.	Gene	Protein function	*M*_w_[kDa]	Score	Coverage
(1)	*lmo0979*	LieA, daunorubicin resistance ATP-binding protein	27.7	334	68.5%
(2)	*lmo0979*	LieA, daunorubicin resistance ATP-binding protein	27.7	286	68.8%

LieA accumulation in the Δ*lftR* mutant is likely explained by repression of *lieAB* transcription through LftR. To test this hypothesis, total RNA preparations of *L. monocytogenes* EGD-e and the Δ*lftR* mutant were probed with a *lieA*-specific riboprobe in a Northern blot experiment. While no transcript signal at all was obtained with wild type RNA, a strong signal corresponding to a single mRNA with a size of roughly 1.6 kb was detected in RNA extracts of the Δ*lftR* mutant (**Figure 3C**). As expected, *lieAB* was not transcribed in the Δ*lftS* mutant, but strongly derepressed in the Δ*lftRS* strain (**Figure 3C**). This clearly demonstrates transcriptional derepression of *lieAB* in the absence of LftR. Furthermore, the observed transcript size of 1.6 kb would be in good agreement with a bicistronic *lieAB* mRNA (**Figure 3D**).

### Repression of *lieAB* Expression through LftR is Crucial for Host Cell Invasion

**FIGURE 4:**
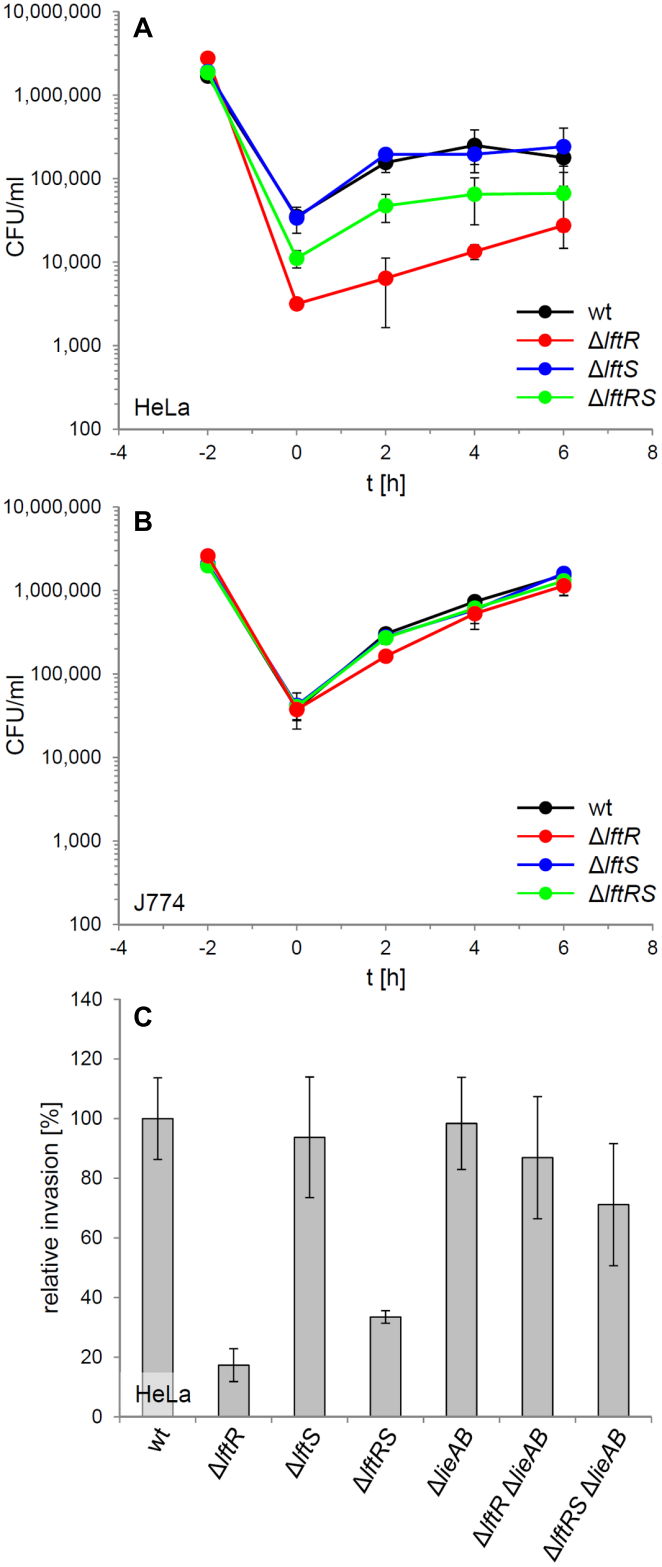
**Attenuation of the *lftRS* mutants in *in vitro* infection experiments. (A)** Intracellular multiplication of strains EGD-e (wt), LMKK42 (Δ*lftR*), LMKK26 (Δ*lftS*), and LMKK31 (Δ*lftRS*) was determined in an *in vitro* infection assay using HeLa cells. The bacterial inoculum was added to the HeLa cells at the time point -2 h, followed by infection for 1 h and killing of extracellular bacteria with gentamycin, in another 1 h incubation step. The number of intracellular multiplying bacteria (CFU/ml) was determined following sampling at 2 h intervals. **(B)** Intracellular growth of the same set of strains in J774 mouse macrophages. **(C)** Invasion of *L. monocytogenes* strains strains EGD-e (wt), LMKK42 (Δ*lftR*), LMKK26 (Δ*lftS*), LMKK31 (Δ*lftRS*), LMS160 (Δ*lieAB*), LMS169 (Δ*lftR* Δ*lieAB*), and LMS168 (Δ*lftRS*Δ*lieAB*) into HeLa cells. Please note that the experiment shown in **(A)** was performed with bacteria grown to stationary phase, whereas the invasion experiment in **(B)** with bacteria from mid-logarithmic growth phase. SD were calculated from experiments performed in triplicate.

The contribution of LftR to virulence of *L. monocytogenes* was tested in an *in vitro* infection assay using HeLa monolayers as host cells. This indicated that the absence of *lftR*, but not that of *lftS* affected invasion into non-phagocytic cells (**Figure 4A**, Supplementary Figure S2D). Interestingly, deletion of *lftS* in the *lftR* mutant suppressed this invasion defect, at least partially. Once inside the HeLa cells, all strains multiplied with an identical rate (**Figure 4A**). Apparently, *lftR* is critical for invasion into non-phagocytic human host cells but not for multiplication inside eukaryotic cells. This conclusion was further substantiated by the observation that all three strains grew similarly inside mouse macrophages (**Figure 4B**). We wondered as to whether the derepression of the putative multidrug resistance transporter encoded by *lieAB* operon might cause this effect, or whether other LftR-dependent factors affect host cell invasion of *L. monocytogenes*. This question was addressed using wild type, Δ*lftR* and Δ*lftRS* strains, in which the entire *lieAB* operon had been removed. Invasion efficiency of the resulting strains LMS160 (Δ*lieAB*), LMS169 (Δ*lftR* Δ*lieAB*) and LMS168 (Δ*lftRS*Δ*lieAB*) was then tested in a separate HeLa cell infection experiment and compared to that of strains EGD-e (wt), LMKK42 (Δ*lftR*), LMKK26 (Δ*lftS*), and LMKK31 (Δ*lftRS*). In good agreement with the previous result, strains lacking *lftR* and *lftRS* showed reduced invasion rates, while invasion efficiency of the Δ*lieAB* mutant was unaffected (**Figure 4C**). Remarkably, the invasion defects associated with lack of *lftR* or *lftRS* genes were suppressed, when the *lieAB* operon was deleted in these strains (**Figure 4C**). This demonstrates that *lftR* contributes to efficient invasion of *L. monocyctogenes* into HeLa cells by preventing overexpression of the putative multidrug resistance transporter encoded by the *lieAB* genes. In contrast to this, introduction of the Δ*lieAB* deletion into the Δ*lftR* background did not restore wild type-like formation of concentric swarming rings in the resulting triple mutant (Supplementary Figure S3A).

### Contribution of the LieAB Transporter to Multidrug Resistance

**FIGURE 5:**
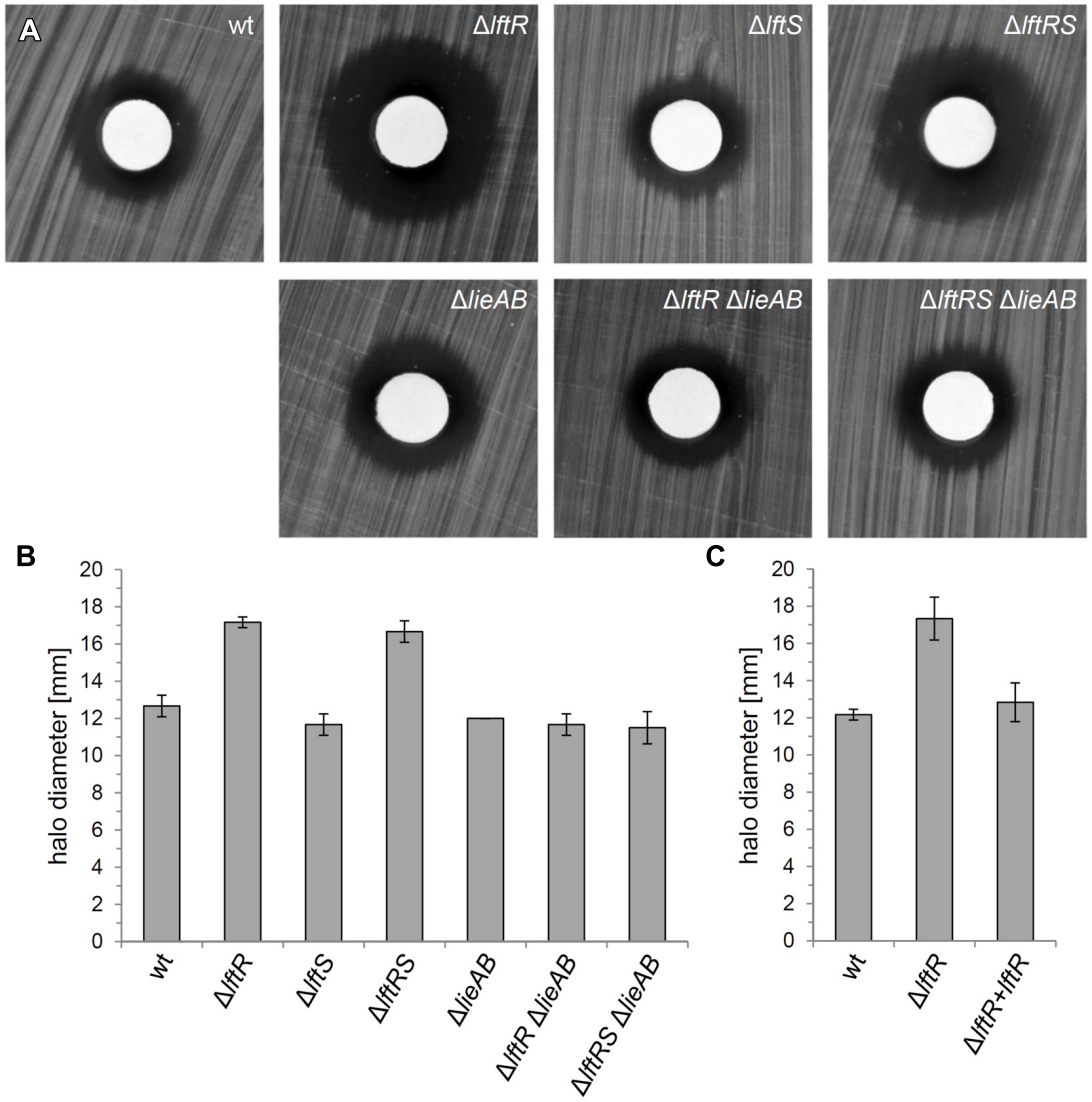
**Reduced resistance of the *L. monocytogenes*Δ*lftR* mutant against ethidium bromide. (A)** Filter disks soaked with 1 mg/ml ethidium bromide solution were put on top of BHI agar plates, which had been inoculated with strains EGD-e (wt), LMKK42 (Δ*lftR*), LMKK26 (Δ*lftS*), LMKK31 (Δ*lftRS*), LMS160 (Δ*lieAB*), LMS169 (Δ*lftR* Δ*lieAB*), and LMS168 (Δ*lftRS*Δ*lieAB*) and incubated overnight at 37°C. **(B)** Quantification of the experiment shown in **(A)**. Diameters of inhibition zones were measured from three independent repetitions and average values and SD are shown. **(C)** Complementation of the Δ*lftR* phenotype in strain LMKK62. Ethidium bromide sensitivity of strains EGD-e (wt), LMKK42 (Δ*lftR*), and LMKK62 (Δ*lftR + lftR*) was determined and quantified as described for the experiment shown in **(A,B)**.

The genes constituting the *lieAB* operon are annotated to encode an ATP-binding subunit and a transmembrane component, respectively, of a daunorubicin (=daunomycin) resistance ABC transporter. This designation was adapted from *Lactococcus lactis* LmrCD, which in fact contributes to resistance against this and a few other compounds, including Hoechst 33342, rhodamine and ethidium bromide (Lubelski et al., 2006). We hypothesized that LftR could contribute to multidrug resistance by controlling expression of the *lieAB* operon and tested this hypothesis by determination of the minimal inhibitory concentrations of several antibiotics with cytoplasmic targets (tetracycline, gentamicin, and chloramphenicol) and vancomycin as a control against Δ*lftR*, Δ*lftS*, Δ*lftRS*, and Δ*lieAB* mutant strains. This revealed the absence of any relevant changes in the susceptibilities of all tested strains against these antibiotics (Supplementary Table S1), indicating that the LieAB transporter does not mediate excretion of these compounds out of the cell. The resistance of the same set of strains against ethidium bromide was tested in a simple disk diffusion assay. A clear increase of the inhibition zones around the compound-soaked filter disks indicated an increased susceptibility of the Δ*lftR* and Δ*lftRS* strains against ethidium bromide (**Figures 5A,B**). In contrast, the Δ*lftS* mutant behaved like wild type, indicating that this was a mere LftR-dependent effect. However, deletion of the *lieAB* operon in the Δ*lftR* and Δ*lftRS* backgrounds corrected their increased susceptibilities against ethidium bromide back to normal wild type levels (**Figures 5A,B**). Moreover, the increased sensitivity of the Δ*lftR* mutant against ethidium bromide was also complemented back to normal wild type levels, when an ectopic allele of *lftR* was expressed in the Δ*lftR* background (strain LMKK62, **Figure 5C**). The susceptibility of the same set of strains against Hoechst 33342 was tested in a similar experimental set-up, but none of the strains was affected (Supplementary Figure S6), indicating specificity of the ethidium bromide effect.

**FIGURE 6:**
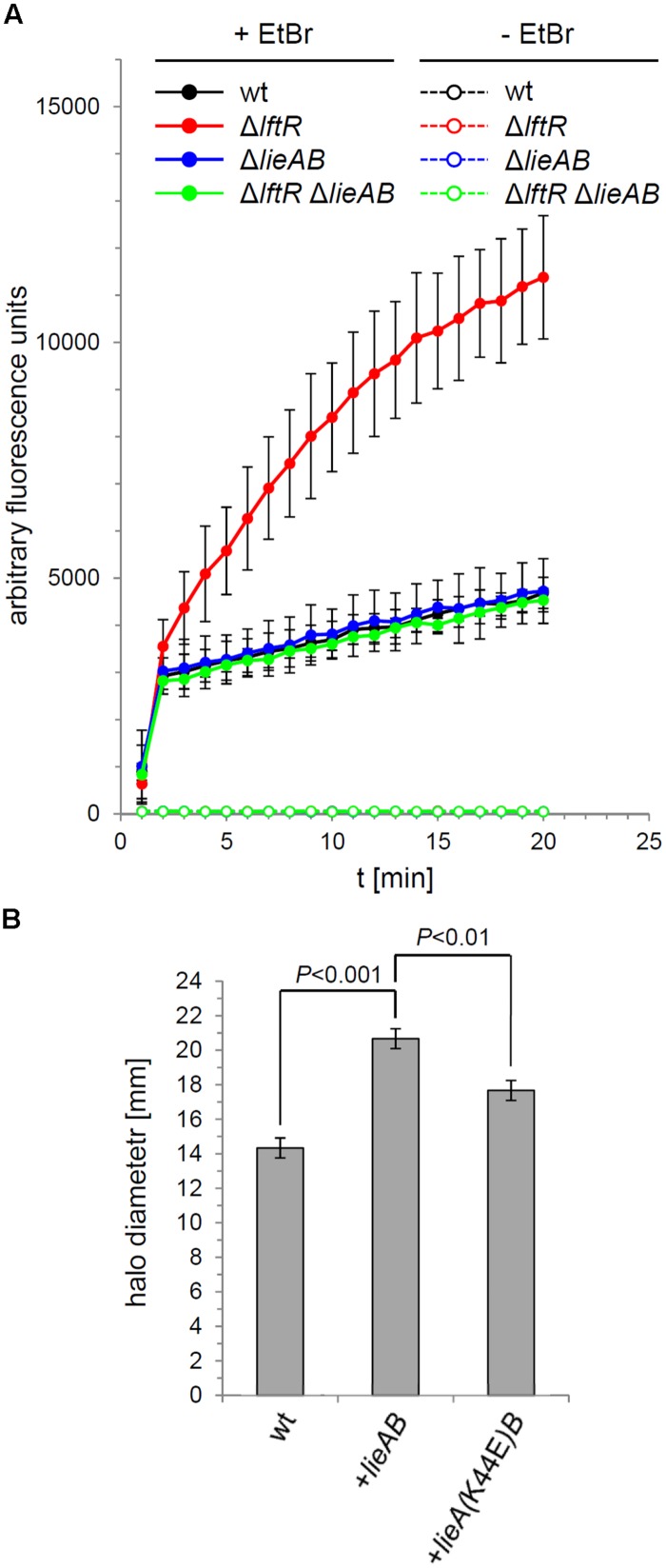
**Ethidium bromide uptake through LieAB. (A)** Intracellular accumulation of ethidium bromide in cells of strains EGD-e (wt), LMKK42 (Δ*lftR*), LMS160 (Δ*lieAB*) and LMS169 (Δ*lftR* Δ*lieAB*) was recorded by measurement of fluorescence over time (see Materials and Methods for details). As a control, the same experiment was performed in the absence of ethidium bromide. Average values and SD were calculated from an experiment performed in triplicate. **(B)** Energy dependence of ethidium bromide uptake by LieAB. Disk diffusion assays were used to test the susceptibility of strains EGD-e (wt), LMNT1 (*+lieAB*), and LMNT2 [*+lieA(K44E)B*] against ethidium bromide (*n* = 3). Significance levels are indicated.

The increased *lieAB*-dependent ethidium bromide susceptibility of the Δ*lftR* mutant suggested that the LieAB transporter possibly facilitates uptake of ethidium bromide into the cell, where it could exert its growth inhibiting effects. In order to test this possibility, uptake of ethidium bromide by the Δ*lftR* mutant cells was recorded by fluorescence measurements over time. This revealed a dramatic increase in ethidium bromide influx into Δ*lftR* cells as compared to the wild type strain (**Figure 6A**). Again, this effect was dependent on the presence of the *lieAB* operon, since its deletion corrected the increased ethidium bromide influx of the Δ*lftR* strain back to the normal wild type situation. This result demonstrates that LieAB acts as an importer, at least when ethidium bromide is considered as a substrate. In good agreement with this conclusion, we observed that artificial overexpression of the *lieAB* operon from an ectopic site was sufficient to increase the susceptibility of *L. monocytogenes* against ethidium bromide (**Figure 6B**). When a mutation was introduced into the ATP-binding site of the ectopically expressed *lieA*, changing the conserved lysine-44 residue into a glutamate, ethidium bromide susceptibility was corrected back, albeit not entirely, to the wild type level (**Figure 6B**). This shows that ethidium bromide influx through LieAB is an energy-dependent process, at least partially.

## Discussion

Invasion of *L. monocytogenes* into eukaryotic host cells is a multifactorial process that requires the concerted action of different attachment and internalization factors, together with the smooth functioning of background house-keeping cellular processes. With respect to LftR, we have identified a so far uncharacterized regulatory protein from the second class of invasion determinants that can disturb this process when deregulated. LftR belongs to the family of PadR-like transcriptional regulators and *L. monocytogenes* EGD-e encodes three more such proteins: LadR (Lmo1408, 26% identity), which represses transcription of the *lmo1409* gene, encoding the multidrug eﬄux pump of the major facilitator-type MdrL, and the uncharacterized Lmo0599 protein (31% identity) (Mata et al., 2000; Huillet et al., 2006). LstR (encoded by the *lmo0422* gene) is involved in heat shock response and was also annotated as a PadR-like transcriptional regulator of *L. monocytogenes* (Supplementary Figure S4B; Zhang et al., 2005).

PadR was first described in *Lactobacillus plantarum* and *Pediococcus pentosaceus* as a repressor of the phenolic acid decarboxylase gene (*padA*), which contributes to detoxification of phenolic acids (Barthelmebs et al., 2000; Gury et al., 2004). In a similar manner, *B. subtilis* PadR represses expression of the phenol acid decarboxylase *padC* gene (Tran et al., 2008). However, the best studied PadR homolog is LmrR from *Lactococcus lactis*, which binds to the promoter regions of its own gene and of the *lmrCD* operon, encoding a heterodimeric multidrug ABC transporter, to repress their transcription (Agustiandari et al., 2008). Crystallography showed that LmrR contains a helix-turn-helix motif for DNA binding and a C-terminal helix for dimerization (Supplementary Figure S4A; Madoori et al., 2009). LmrR binds to an inverted repeat in the promoter regions of the *lmrR* and *lmrCD* genes with the consensus sequence ATGT-N_8_-ACAT. Identical binding motifs were also described for PadR from *P. pentosaceus* and *L. plantarum* as well as for *L. monocytogenes* LadR (Gury et al., 2004; Huillet et al., 2006; Agustiandari et al., 2011). Binding of compounds like Hoechst 33342, daunomycin or ethidium is thought to induce conformational changes in LmrR, which prevent promoter recognition and thus lead to induction of the *lmrR* and *lmrCD* genes (Madoori et al., 2009; Agustiandari et al., 2011; Takeuchi et al., 2014). Most likely, LftR senses similar compounds.

LmrR does not seem to be the real LftR equivalent of *Lactococcus lactis*, since a higher degree of identity to LftR is observed with another, yet uncharacterized *Lactococcus lactis* transcriptional regulator (encoded by the *llmg_2339* gene). Likewise, the listerial *lieAB* operon is only similar to but does not correspond to *Lactococcus lactis lmrCD*, which in turn shares the highest similarity with the *lmo2751–2752* genes, coding for another putative multidrug ABC transporter of *L. monocytogenes*. Thus, the *lftR lieAB* genes are similar but not identical with the *lmrR lmrCD* module of *Lactococcus lactis*.

It is not clear, whether LftR represses transcription of *lieAB* genes directly, or whether this is an indirect effect. Sequence searches have not identified the typical ATGT-N_8_-ACAT PadR binding motif in the *lmo0979–0980* promoter region or in front of the *lftR* gene. Also, a comparison of both promoters did not uncover another putative LftR binding site. Rather, an ideal ATGT-N_8_-ACAT motif is present in the P*_mdrL_* promoter, where it represents the binding site for LadR (Huillet et al., 2006). There is some degree of sequence variation in the DNA binding helices of the PadR proteins (Supplementary Figures S4A,B), so the DNA binding site for LftR might differ from the canonical PadR binding site. Alternatively, up-regulation of *lieAB* expression in the absence of LftR could be an indirect effect. We have looked for PadR binding sites in the whole *L. monocytogenes* genome and identified 68 matches in total, out of which 16 lie within a 400 bp range upstream of genes. However, these putative PadR sites overlap with potential promoter sequences only in a few cases (Supplementary Table S2). Among these are the promoters of the *lmo0018* gene (encoding a β-galactosidase), the promoter of the *lmo0748–0751* operon and the P*_mdrL_* promoter itself (Supplementary Table S2). The β-galactosidase encoded by *lmo0018* is homologous to *B. subtilis* BglH (62% identity), which is necessary for catabolism of aryl-β-glycosides (Le Coq et al., 1995). In contrast, the *lmo0748–0751* operon mainly encodes uncharacterized *Listeria*-specific genes with an interesting exception: the second gene of the operon (*lmo0749*) is annotated as a transcriptional regulator of the Cro-family of phage proteins, suggesting that LftR and/or other PadR-type proteins could be part of a hierarchical network of transcriptional regulators.

While PadR-type regulators such as LftR can be found in many bacteria, homologs of LftS are only present in the *Listeria* and some (but not all) *Bacillus, Paenibacillus, Lactococcus*, and *Enterococcus* species, in some *Clostridia* and a few actinobacterial species. Deletion of *lftS* alone has not resulted in any conspicuous phenotype in our hands. However, removal of *lftS* suppressed the swarming defect and (partially) the invasion defect of the Δ*lftR* mutant. If LftR – like LmrR of *Lactococcus lactis* – auto-represses transcription of the *lftRS* operon, then *lftS* would be de-repressed in the absence of *lftR*. Consequently, deletion of *lftS* in a Δ*lftR* mutant would remove all phenotypes that result from derepression of LftS. With this logic, we observe two classes of effects upon *lftR* deletion: LftS-dependent phenotypes that result from derepression of LftS. Such an effect is observed with the Δ*lftR* swarming phenotype, which is corrected back to wild type levels in a Δ*lftRS* double mutant. In contrast, the increased ethidium bromide sensitivity of the Δ*lftR* mutant constitutes a second class of effect, which is LftS-independent and merely caused by derepression of the LieAB transporter. The combination of both effects explains the invasion defect of the Δ*lftR* mutant, which is mainly caused by *lieAB* derepression, but which is also partially rescued by deletion of *lftS*. Presently, it is not clear, how LftS could contribute to invasion or concentric swarming ring formation, but the effect it exerts must be LftR-independent.

The pronounced invasion defect that results from the derepression of the *lieAB* operon in the Δ*lftR* mutant suggests that substrates of this transporter are detrimental for the infection process. There are 71 more *lieA*-like genes encoding ATP-binding proteins of ABC transporters present in the *L. monocytogenes* EGD-e genome, but the membrane component encoded by *lieB* is unique. The fact that multidrug resistance transporters are critical players in the infection process is not unprecedented in *L. monocytogenes*. Mdr transporters of the major facilitator type mediate extrusion of cyclic-di-AMP and this contributes to induction of the immune response in macrophages (Crimmins et al., 2008; Woodward et al., 2010; Kaplan Zeevi et al., 2013; Tadmor et al., 2014). Whether the LieAB transporter is a mere importer or whether it also excretes compounds is presently not clear. Its genuine substrate with relevance for invasion is also not known and ethidium bromide clearly has to be considered an artificial substrate. It is tempting to speculate, that uptake of natural LieAB substrates might be advantageous for *L. monocytogenes* during life as an environmental saprophyte, but disadvantageous during growth in rich media or during infection. Earlier studies have shown that *lftR* expression is upregulated during stationary phase, suggesting that even LftR levels are subject to control (Chatterjee et al., 2006; Toledo-Arana et al., 2009). Tightly controlled conditional expression of *lieAB* could adjust the LieAB transporter levels to the respective growth condition.

## Conflict of Interest Statement

The authors declare that the research was conducted in the absence of any commercial or financial relationships that could be construed as a potential conflict of interest.
