# Supplementary material for: The PadR-like transcriptional regulator LftR ensures efficient invasion of Listeria monocytogenes into human host cells
Source: Front Microbiol. 2015 Jul 28;6:772. doi: 10.3389/fmicb.2015.00772 (PMC4517056; doi:10.3389/fmicb.2015.00772)
Supplement: Supplementary file 1 [file Presentation_1.PDF]

Supplementary Information to:

The PadR-like transcriptional regulator LftR ensures efficient invasion of *Listeria monocytogenes* into human host cells

Karan Gautam Kaval, Birgitt Hahn, Nayana Tusamda, Dirk Albrecht and Sven Halbedel

Table S1: Antibiotic susceptibilities of *L. monocytogenes lftRS* and *lieAB* mutants.

Table S2: Presence of PadR binding sites in front of *L. monocytogenes* EGD-e genes.

Figure S1: The *secA2G484E* mutation causes the rough phenotype.

Figure S2: Phenotype of the reconstituted *lftS::Tn* transposon insertion mutant.

Figure S3: Deletion of *lieAB* does not suppress the swarming phenotype of the Δ*lftR* mutant.

Figure S4: LftR is a transcriptional regulator of the PadR family.

Figure S5: Relief of LieA overexpression upon deletion of the *lieAB* operon.

Figure S6: Cytotoxicity of Hoechst 33342 on *L. monocytogenes lftRS* and *lieAB* mutant strains.

Table S1: Antibiotic susceptibilities of *L. monocytogenes* mutants lacking the *lftRS* and *lieAB* genes.

		Minimal inhibitory concentration (µg/ml) [a]			
**Strain**	**Genotype**	**Tetracycline**	**Gentamicin**	**Chloramphenicol**	**Vancomycin**
EGD-e	wt	0.14±0.05	0.29±0.18	2.00±1.00	1.17±0.29
LMKK42	Δ*lftR*	0.14±0.05	0.23±0.13	3.00±1.00	1.33±0.29
LMKK26	Δ*lftS*	0.17±0.04	0.29±0.18	2.33±0.58	0.92±0.14
LMKK31	Δ*lftRS*	0.19±0.00	0.38±0.13	3.00±0.00	1.08±0.39
LMS160	Δ*lieAB*	0.13±0.00	0.29±0.19	2.33±0.58	0.96±0.56
LMS169	Δ*lftR* Δ*lieAB*	0.15±0.04	0.25±0.11	2.17±0.77	1.17±0.29
LMS168	Δ*lftRS* Δ*lieAB*	0.13±0.00	0.27±0.20	2.17±0.77	1.33±0.29

[a] Antibiotic susceptibilities are expressed as average values of minimal inhibitory concentrations ± standard deviations calculated from three independent experiments.

Table S2: Presence of PadR binding sites (ATGT-N_8-ACAT) in front of *L. monocytogenes* EGD-e genes.

position[1]	strand	gene	function	sequence[2]
-65	+	*lmo0018*	beta-glucosidase	GCTTGCT**ATGTAATGCATTACAT**ATTCTATAGTGGAAAAGTAAATTATATTCGTGAGA
-226	+	*lmo0325*	similar to transcriptional regulators	AAAGAGA**ATGTGAAAAATGACAT**TCTTTTTTTGTGCTATTAATACTTAAAAAAGGAAG
-182	+	*lmo0379*	unknown	ACTTAGT**ATGTTGGTTGTTACAT**CTGAGAATGAATTAAATGATAGTGGAAAAGTCTTA
-96	+	*lmo0748*	unknown	CTATTAT**ATGTAAAATAAAACAT**TTTTTATGTCAAATATATTTGACATAATGTAAAGT
-279	+	*lmo1276*	Gid, glucose inhibited division protein A, tRNA (uracil-5-)-methyltransferase	GTCCAAA**ATGTGAGAAAGGACAT**GTAATAGAAAGAAAAAGTAAGAAAAAACGTATTTT
-79	+	*lmo1409*	MdrL	GTTATAT**ATGTAAATATTGACAT**TAGTATTTATCAAGCGTAATATAATAATTGAGTTT
-292	-	*lmo1420*	MurB, UDP-N-acetylenolpyruvoylglucosamine reductase	GTCTTTT**ATGTCCAAAGTTACAT**TATTTACTGCTGCTGTTTTATCATCGCTGTATTTT
-298	+[3]	*lmo1635*	unknown	GGTAAAG**ATGTTGGTATCGACAT**GAGTCTTAAAGGACAAAATGTTGCTAAAAAAGATT
-53	-[4]	*lmo1654*	putative cell surface protein	TGACAAT**ATGTTTGAGGCTACAT**TTTATTAATTATTACATACAAAGAGGGAGAGCGTA
-343	-	*lmo1696*	unknown	CTATGTA**ATGTTTCCGCAAACAT**AGCAATATTTCCTGTAAAAAAATAAGTAACACCTT
-345	-	*lmo1744*	unknown	CATAATA**ATGTTTTCCTACACAT**TGATACGAACACACTTACACATTTAGAAGATAAAA
-353	-	*lmo1850*	MarR family transcriptional regulator	ACGCCAG**ATGTGGTTGTGAACAT**GCCAGATTATGCAACTATGACTATCCCTTCCTCCA
-325	+	*lmo2218*	unknown	TTAAAAG**ATGTTTCTTCTGACAT**CATGGAATCTGTTTCCAAATGGAATGAAGAAATGG
-150	-	*lmo2523*	similar to *B. subtilis* single-strand DNA-binding protein SsbB	GCTTTGG**ATGTAATTACATACAT**AAAATGATATTTTTCTCTTATGAGAAAATAACCTT
- 68	-[5]	*lmo2710*	unknown	AAAAATA**ATGTATAATGCTACAT**AAACTGGTACAATATAAAATAGTAAAGCAATTCAT

[1] Distance in bp from start codon of a gene.

[2] Promoter sequences have been deduced from transcriptional start points given by Wurtzel *et al.* (1). -35 regions are boxed and underlined with a dashed line, -10 regions are boxed. PadR sites are shown in bold letters and its inverted repeats are shaded in grey

[3] The PadR site of *lmo1635* is in the C-terminus of the *lmo1634* gene.

[4] The PadR site of *lmo1654* is in 5′-UTR of the *lmo1654-1653* transcript.

[5] The PadR site of *lmo2710* is in 5′-UTR of the *lmo2710* transcript.

REFERENCES

1. **Wurtzel O, Sesto N, Mellin JR, Karunker I, Edelheit S, Becavin C, Archambaud C, Cossart P, Sorek R.** 2012. Comparative transcriptomics of pathogenic and non-pathogenic *Listeria* species. Molecular systems biology **8:**583.

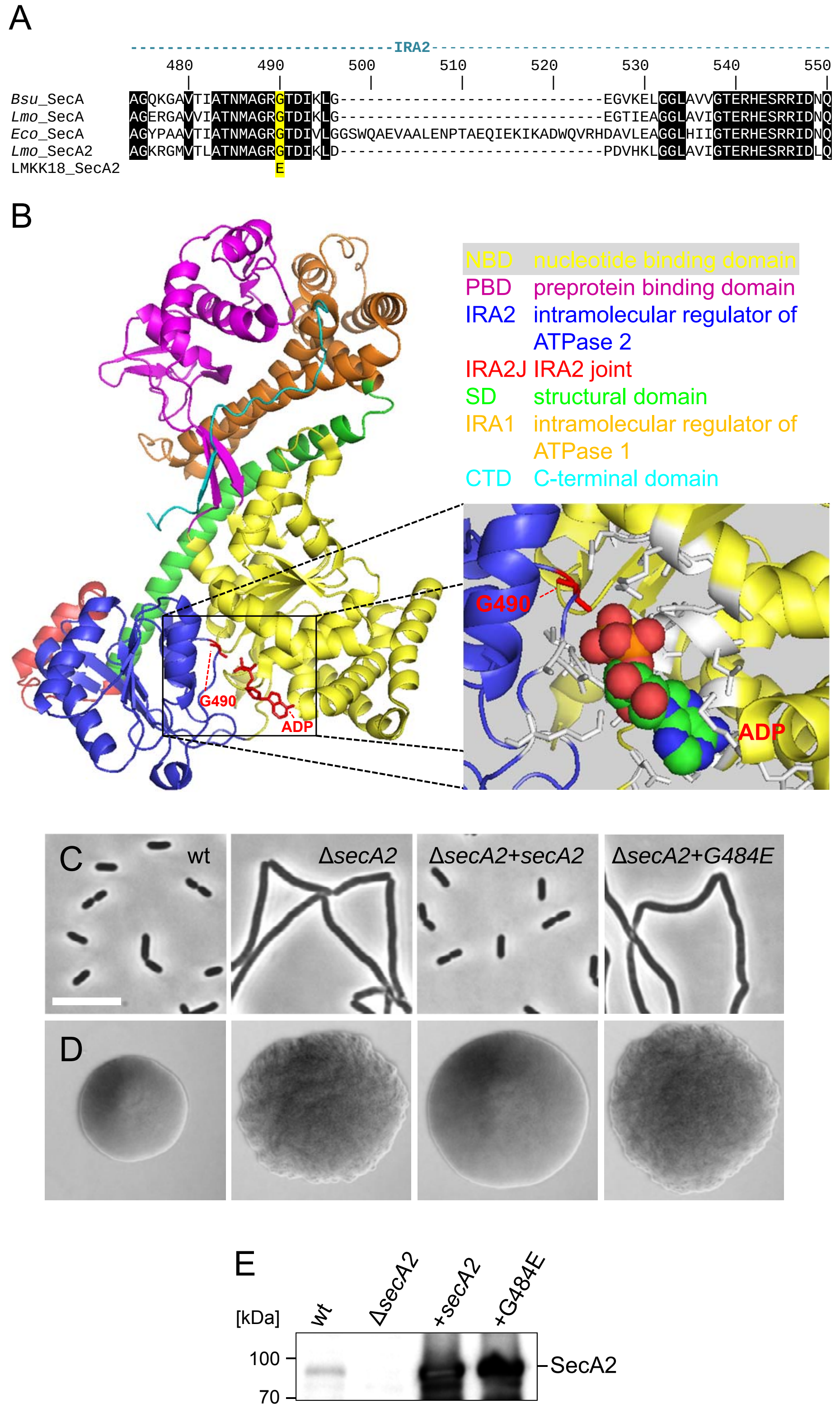

Fig. S1: The *secA2G484E* mutation causes the rough phenotype.
(A) Multiple alignment of SecA proteins from *B. subtilis* (*Bsu*), *E. coli* (*Eco*) and *L. monocytogenes* (*Lmo*) with *L. monocytogenes* SecA2 showing a section of their IRA2 domains. Residues are numbered according to the sequence of *B. subtilis* SecA. G490 of *B. subtilis* SecA corresponds to G484 in *L. monocytogenes* SecA2, which is mutated in strain LMKK18 and replaced by a glutamate residue. (B) Crystal structure of *B. subtilis* SecA in complex with ADP (red) (PDB ID 1TF2) xxx(46). Domains are colored as indicated and the G490 side chain is marked in red. The nucleotide binding pocket is shown at a higher magnification with all relevant side chains colored in white, except G490, which is marked in red. ADP is shown as a space-filling model. (C) Phase contrast micrographs showing morphology of *L. monocytogenes* strains EGD-e (wt), LMS81 (Δ*secA2*), LMKK27 (Δ*secA2* + *secA2*) and LMKK28 (Δ*secA2* + *secA2G484E*) cultivated to mid-logarithmic growth phase in BHI broth at 37°C. (D) Colony morphology of the same set of strains after growth on BHI agar plates at 37°C. Images of the colonies were documented directly from the plate using 10-fold magnification. (E) Western blot showing expression of SecA2 in the same set of strains.

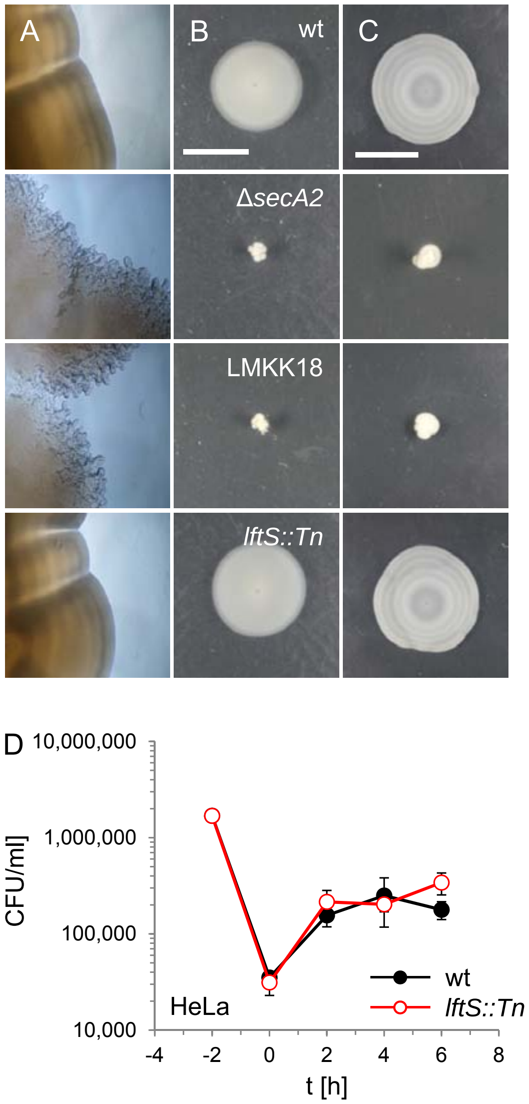

Fig. S2: Phenotype of the reconstituted *lftS::Tn* transposon insertion mutant.
(A) Micrographs illustrating colony morphology of *L. monocytogenes* strains EGD-e (wt), LMS81 (Δ*secA2*), LMKK18 (*lftS::Tn secA2G484E*), and LMKK64 (*lftS::Tn*). Strains were streaked on BHI agar plates, incubated at room temperature for 72 h and were directly documented from the plate by light microscopy.(B-C) Swarming assay for the same set of strains. Soft LB agar plates were stab inoculated with the mentioned strains, incubated at 30°C for 24 h and then documented (B). The same plates photographed after 6 days of incubation at room temperature (C). Scale in A indicates 1 cm, while that in B indicates 4 cm. (D) HeLa cell infection experiment with strains EGD-e (wt) and LMKK64 (*lftS::Tn*).

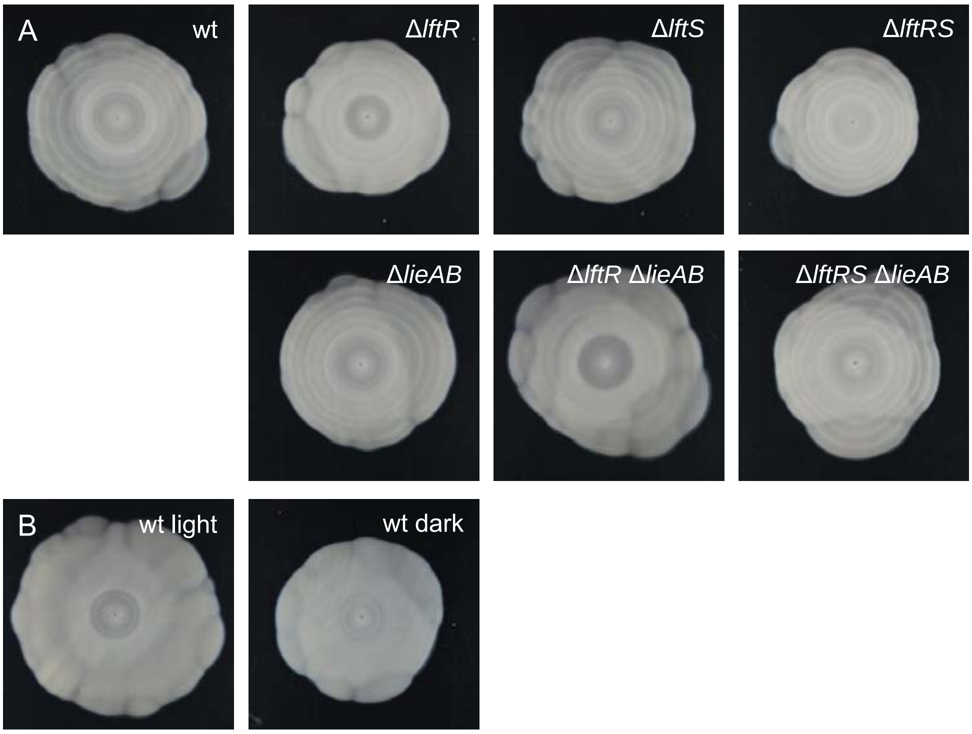

Fig. S3: Deletion of *lieAB* operon does not suppress the swarming phenotype of the Δ*lftR* mutant.
(A) Concentric swarming halo formation of strains lacking *lftR* (LMKK42), *lftS* (LMKK26), *lftRS* (LMKK31), *lieAB* (LMS160) and combinations thereof (Δ*lftRS* Δ*lieAB,* LMS168; Δ*lftR* Δ*lieAB,* LMS169). Strains were stab-inoculated into LB soft agar plates and incubated at 30°C for 24 h in the dark and then for 6 days at room temperature under ambient light conditions. (B) *L. monocytogenes* strain EGD-e (wt) was incubated for 24 h at 30° in the dark and then at room temperature for 6 consecutive days either in the dark or exposed to constant light.

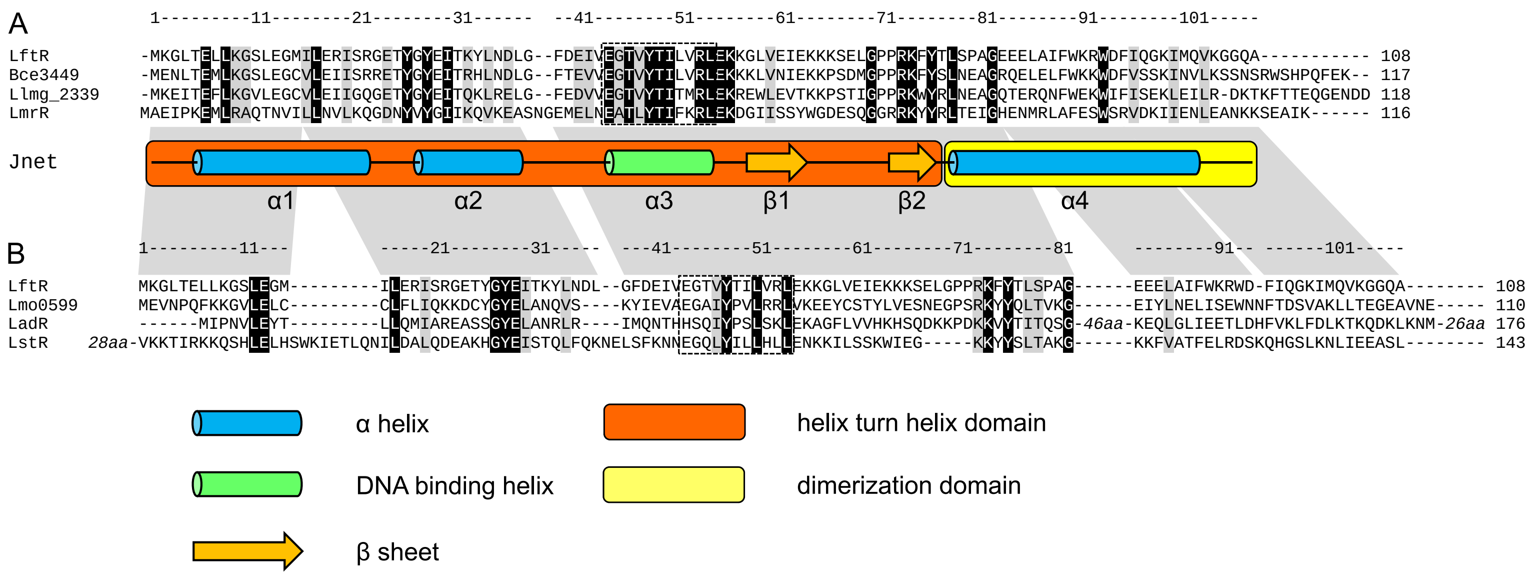

Fig. S4: LftR is a transcriptional regulator of the PadR family.
(A) Multiple sequence alignment of LftR and its homologs Bce3449 from *B. cereus* ATCC 10987 and LmrR as well as Llmg_2339 from *Lactococcus lactis* subsp. *cremoris* MG1363. The result of a Jnet secondary structure prediction is given below the alignment and domains are indicated. The DNA binding helix is boxed. (B) Multiple sequence alignment of PadR-type transcriptional regulators encoded by *L. monocytogenes* strain EGD-e. Identical amino acid positions are indicated by black boxes, similar positions are shaded grey. Numbering of amino acid positions is according to the LftR protein sequence.

Kaval *et al.*, Fig. S4

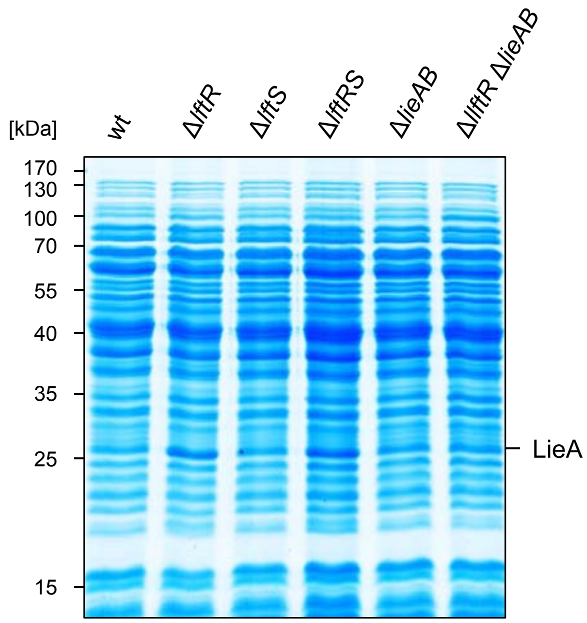

Fig. S5: Relief of LieA overexpression upon deletion of the *lieAB* operon.
L. monocytogenes strains EGD-e (wt), LMKK42 (Δ*lftR*), LMKK26 (Δ*lftS*), LMKK31 (Δ*lftRS*), LMS160 (Δ*lieAB*) and LMS169 (Δ*lftR* Δ*lieAB*) were grown in BHI broth at 37°C to an OD_{600} of 1.0, total cellular proteins were isolated and separated by SDS-PAGE. The position of LieA is indicated.

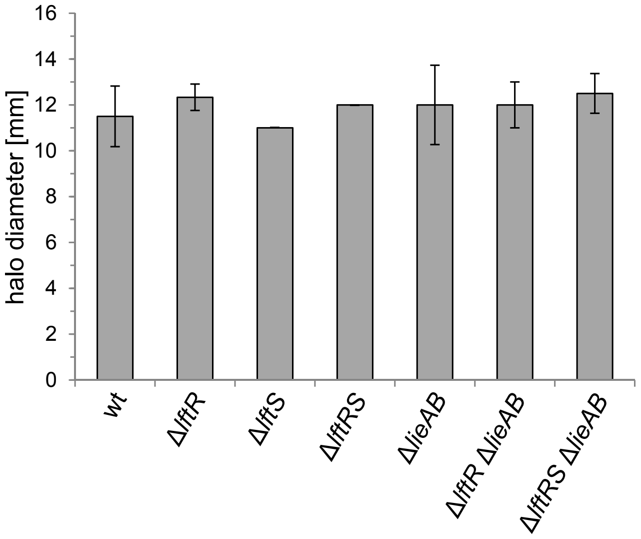

Fig. S6: Cytotoxicity of Hoechst 33342 on *L. monocytogenes lftRS* and *lieAB* mutant strains
Sensitivity of *L. monocytogenes* strains EGD-e (wt), LMKK42 (Δ*lftR*), LMKK26 (Δ*lftS*), LMKK31 (Δ*lftRS*), LMS160 (Δ*lieAB*) and LMS169 (Δ*lftR* Δ*lieAB*) against Hoechst 33342 was tested in a disc diffusion assay as described in the Material and Methods section. Average values of halo diameters and standard deviations were calculated from experiments performed in triplicate.
